# Boarder control on the IFT train

**DOI:** 10.7554/eLife.02531

**Published:** 2014-03-18

**Authors:** Cécile Fort, Philippe Bastin

**Affiliations:** 1 **Cécile Fort** is at the Trypanosome Cell Biology Unit, Institut Pasteur & CNRS, Paris, France; 2 **Philippe Bastin** is at the Trypanosome Cell Biology Unit, Institut Pasteur & CNRS, Paris, Francepbastin@pasteur.fr

**Keywords:** chlamydomonas, flagella, dynein, axoneme, flagellar growth, zebrafish

## Abstract

New details are revealed about the system that transports proteins to the tip of flagella during growth.

**Related research article** Ishikawa H, Ide T, Yagi T, Jiang X, Hirono M, Sasaki H, Yanagisawa H, Wemmer KA, Stainier DYR, Qin H, Kamiya R, Marshall WF. 2014. TTC26/DYF13 is an intraflagellar transport protein required for transport of motility-related proteins into flagella. *eLife* **3**:e01566. doi: 10.7554/eLife.01566

**Image** Immunofluoresence image of a wild-type green alga showing (in red) proteins inside both flagella

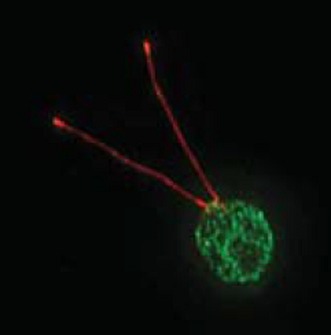

Cilia and flagella are cylindrical organelles that are present at the surface of many eukaryotic cells, where they detect changes in the local environment and—when they beat—help the cells to move. An individual cilium or flagellum grows by adding new protein subunits to its tip, using a special mechanism to move proteins from the body of the cell to the tip of the organelle.

Intraflagellar transport, or IFT, was discovered by Joel Rosenbaum and co-workers at Yale University twenty years ago while they were studying the green alga *Chlamydomonas*, which is a classic model for flagellum studies (Kozminski et al., 1993). In this form of transport, complexes containing about 20 IFT proteins are moved from the base to the tip of the flagella, and are then recycled back towards the base. This movement can be compared to trains travelling on microtubule tracks. At the time, it was proposed that the cargoes, or passengers, on the IFT ‘train’ are the precursors of the axoneme that forms the core of the flagellum (Figure 1A). This very reasonable hypothesis is supported by the observation that construction of the flagellum is inhibited if a single IFT protein is missing (Pazour et al., 2000).

**Figure 1.**
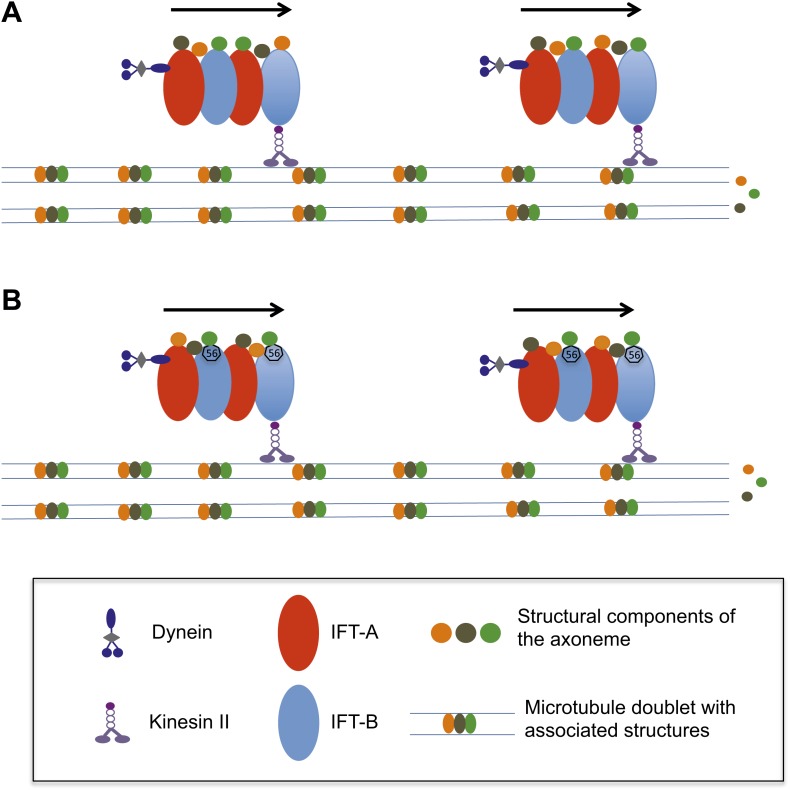
How does the axoneme at the core of a flagellum grow? (**A**) The precursor proteins that will become part of the axoneme are loaded onto the IFT trains. These trains are formed of tightly bound IFT proteins (red and blue ovals) and are powered towards the end of the flagellum by kinesin II molecular motors. The precursor proteins (orange, brown and green circles) are randomly distributed within the IFT trains. (**B**) According to the new findings by Ishikawa et al., a protein called IFT56 (shown here as a black heptagon) could function as a specific adaptor ensuring transport of a limited but specific subset of axoneme components (in this case, the green one).

However, demonstrating the presence of passengers on IFT trains turned out to be very tricky. IFT trains were purified from different organisms, confirming that IFT proteins were tightly bound together, but the presence of cargoes could not be shown convincingly. This suggests that association is transitory and cannot survive biochemical purification. So are the passengers hiding?

Now, in *eLife*, Hiroaki Ishikawa and Wallace Marshall, both from the University of California, San Francisco, and co-workers in the US, Japan and Germany report on a new IFT protein called IFT56 (also known as DYF13, TTC26B or PIFTC3) that could deliver a specific set of proteins that power the movement of flagella (Ishikawa et al., 2014). IFT56 would therefore function as a train conductor selecting particular proteins to board the train (Figure 1B). This exciting proposal is based on exhaustive analysis of cilia and flagella in zebrafish and *Chlamydomonas* when IFT56 expression was prevented. A mutation leading to the production of a severely truncated IFT56 protein did not interfere with train speed or frequency, but resulted in the formation of slightly shorter flagella with reduced motility. Proteomic analyses revealed that these flagella contained reduced amounts of several proteins associated with the generation or control of flagellum beating. Although the model is based on indirect evidence, the recent report that cargo proteins can finally be visualised (Wren et al., 2013) means that it is now possible to test this hypothesis: in other words, we will be able to unmask the passengers.

If IFT56 really acts as a conductor, how does it function? Recent data indicate that only a minority of the trains transport cargoes (Wren et al., 2013), despite the presence of IFT56 on all of them. So what controls loading? Is it simply the availability of cargoes or are some proteins marked in some way to indicate that they should be sent to the flagellum? In other words, do passengers need tickets to gain access to the train? This ticket could be a single post-translational modification such as phosphorylation.

The absence of IFT56 affects different organisms in different ways. In protozoa called trypanosomes, an absence of IFT56 causes flagella to go missing (Absalon et al., 2008; Franklin and Ullu, 2010), but in zebrafish (Zhang et al., 2012) and the green alga it only results in shorter flagella. This absence could impact the stability or movement of the IFT train. Perhaps in some species, the conductor is also an engineer, assisting train formation and function.

Alternatively, such a difference could reflect how the stability of the flagellum depends on the elements that power flagellar beating. For example, in *Leishmania*, the cousins of trypanosomes, a modification to the molecular motor results in the construction of much shorter flagella (Harder et al., 2010). This same modification in *Chlamydomonas* does not have this effect (Kamiya, 1988). In this case, the different phenotypes would be due to the nature of the flagellum itself–IFT56 and the IFT train would not play a direct role in determining them.

Intriguingly, IFT56 is also associated with IFT trains in immotile cilia that do not possess the motility elements discussed above (Blacque et al., 2005). This may seem to contradict the model proposed by Ishikawa et al. but could be explained by the conductor specialising to detect and transport any cargoes that possess the same ticket. In these conditions, IFT56 could ship very different protein complexes providing a single recognition element is shared between them.

Twenty years after the discovery of intraflagellar transport, we are now getting the first insights about putative cargoes. In the future, progress in live imaging, functional genomics and better understanding of the structure of IFT trains should illuminate the mechanisms by which cargoes are recognised, loaded and delivered to their destination.

## References

[bib1] Absalon S Blisnick T Kohl L Toutirais G Dore G Julkowska D Tavenet A Bastin P 2008 Intraflagellar transport and functional analysis of genes required for flagellum formation in trypanosomes. Molecular Biology of the Cell 19:929–944. 10.1091/mbc.E07-08-0749 18094047 PMC2262991

[bib2] Blacque OE Perens EA Boroevich KA Inglis PN Li C Warner A Khattra J Holt RA Ou G Mah AK McKay SJ Huang P Swoboda P Jones SJ Marra MA Baillie DL Moerman DG Shaham S Leroux MR 2005 Functional genomics of the cilium, a sensory organelle. Current Biology 15:935–941. 10.1016/j.cub.2005.04.059 15916950

[bib3] Franklin JB Ullu E 2010 Biochemical analysis of PIFTC3, the *Trypanosoma brucei* orthologue of nematode DYF-13, reveals interactions with established and putative intraflagellar transport components. Molecular Microbiology 78:173–186. 10.1111/j.1365-2958.2010.07322.x 20923419 PMC3010206

[bib4] Harder S Thiel M Clos J Bruchhaus I 2010 Characterization of a subunit of the outer dynein arm docking complex necessary for correct flagellar assembly in *Leishmania donovani*. PLOS Neglected Tropical Diseases 4:e586. 10.1371/journal.pntd.0000586 20126266 PMC2811169

[bib5] Ishikawa H Ide T Yagi T Jiang X Hirono M Sasaki H Yanagisawa H Wemmer KA Stainier DYR Qin H Kamiya R Marshall WF 2014 TTC26/DYF13 is an intraflagellar transport protein required for transport of motility-related proteins into flagella. eLife 3:e01566. 10.7554/eLife.01566 24596149 PMC3936282

[bib6] Kamiya R 1988 Mutations at twelve independent loci result in absence of outer dynein arms in Chylamydomonas reinhardtii. The Journal of Cell Biology 107:2253–2258. 10.1083/jcb.107.6.2253 2974040 PMC2115671

[bib7] Kozminski KG Johnson KA Forscher P Rosenbaum JL 1993 A motility in the eukaryotic flagellum unrelated to flagellar beating. Proceedings of the National Academy of Sciences of the United States of America 90:5519–5523. 10.1073/pnas.90.12.5519 8516294 PMC46752

[bib8] Pazour GJ Dickert BL Vucica Y Seeley ES Rosenbaum JL Witman GB Cole DG 2000 Chlamydomonas IFT88 and its mouse homologue, polycystic kidney disease gene Tg737, are required for assembly of cilia and flagella. The Journal of Cell Biology 151:709–718. 10.1083/jcb.151.3.709 11062270 PMC2185580

[bib9] Wren KN Craft JM Tritschler D Schauer A Patel DK Smith EF Porter ME Kner P Lechtreck KF 2013 A differential cargo-loading model of ciliary length regulation by IFT. Current Biology 23:2463–2471. 10.1016/j.cub.2013.10.044 24316207 PMC3881561

[bib10] Zhang Q Liu Q Austin C Drummond I Pierce EA 2012 Knockdown of ttc26 disrupts ciliogenesis of the photoreceptor cells and the pronephros in zebrafish. Molecular Biology of the Cell 23:3069–3078. 10.1091/mbc.E12-01-0019 22718903 PMC3418303

